# Efficient engraftment of pluripotent stem cell-derived myogenic progenitors in a novel immunodeficient mouse model of limb girdle muscular dystrophy 2I

**DOI:** 10.1186/s13395-020-00228-3

**Published:** 2020-04-22

**Authors:** Karim Azzag, Carolina Ortiz-Cordero, Nelio A. J. Oliveira, Alessandro Magli, Sridhar Selvaraj, Sudheer Tungtur, Weston Upchurch, Paul A. Iaizzo, Qi Long Lu, Rita C. R. Perlingeiro

**Affiliations:** 1 Lillehei Heart Institute, Department of Medicine, University of Minnesota, 4-128 CCRB, 2231 6th St. SE, Minneapolis, MN 55455 USA; 2 Department of Integrative Biology and Physiology, University of Minnesota, Minneapolis, MN USA; 3 Stem Cell Institute, University of Minnesota, Minneapolis, MN USA; 4 Visible Heart Laboratories, Department of Surgery, University of Minnesota, Minneapolis, MN USA; 5 McColl-Lockwood Laboratory for Muscular Dystrophy Research, Cannon Research Center, Carolinas Medical Center, Atrium Health, Charlotte, North Carolina, NC USA

**Keywords:** Muscular dystrophy, FKRP, LGMD2I, Pluripotent stem cells, Transplantation, Muscle regeneration

## Abstract

**Background:**

Defects in α-dystroglycan (DG) glycosylation characterize a group of muscular dystrophies known as dystroglycanopathies. One of the key effectors in the α-DG glycosylation pathway is the glycosyltransferase fukutin-related protein (FKRP). Mutations in *FKRP* lead to a large spectrum of muscular dystrophies, including limb girdle muscular dystrophy 2I (LGMD2I). It remains unknown whether stem cell transplantation can promote muscle regeneration and ameliorate the muscle wasting phenotype associated with *FKRP* mutations.

**Results:**

Here we transplanted murine and human pluripotent stem cell-derived myogenic progenitors into a novel immunodeficient *FKRP*-mutant mouse model by intra-muscular injection. Upon both mouse and human cell transplantation, we observe the presence of donor-derived myofibers even in absence of pre-injury, and the rescue of α-DG functional glycosylation, as shown by IIH6 immunoreactivity. The presence of donor-derived cells expressing Pax7 under the basal lamina is indicative of satellite cell engraftment, and therefore, long-term repopulation potential. Functional assays performed in the mouse-to-mouse cohort revealed enhanced specific force in transplanted muscles compared to PBS-injected controls.

**Conclusions:**

Altogether, our data demonstrate for the first time the suitability of a cell-based therapeutic approach to improve the muscle phenotype of dystrophic *FKRP*-mutant mice.

## Background

Muscular dystrophies (MDs) are a group of genetic diseases characterized by progressive degeneration and muscle weakness. Among them, dystroglycanopathies represent a significant subgroup, which is characterized by hypoglycosylation of α-dystroglycan (DG; OMIM 128239) [1]. α-DG is a key effector of the dystrophin glycoprotein complex as it ensures the binding of the actin cytoskeleton to the extracellular matrix (ECM) [2], however, this binding requires functionally glycosylated α-DG. α-DG glycosylation is composed of both N-linked and O-linked glycans, with the latter mediating the binding of α-DG to ECM proteins, such as laminin, agrin, perlecan, neurexin, and pikachurin [3–8]. Therefore, hypoglycosylation of α-DG in muscle leads to reduced α-DG binding to the ECM, fragile sarcolemma, and ultimately to the dystrophic phenotype [9].

At present, 18 genes involved in the α-DG glycosylation pathway have been linked to dystroglycanopathies [10], including the fukutin-related protein (FKRP; OMIM 606596). FKRP is a ribitol-phosphate transferase that utilizes CDP-ribitol as a substrate to add a ribitol-phosphate into the glycosylation chain, a critical step to generate functionally glycosylated α-DG [11, 12]. *FKRP* mutations are associated with a large spectrum of dystroglycanopathies from severe forms, such as congenital muscular dystrophy and Walker–Warburg syndrome, to limb girdle muscular dystrophy type 2I (LGMD2I) [13–15]. As in other types of MDs, only palliative treatments are currently available for these patients.

Several promising approaches are being investigated to restore functional glycosylation of α-DG using available *FKRP*-mutant mouse models [16–22]. One strategy is to increase the levels of metabolites involved in the FKRP-mediated α-DG glycosylation process, such as ribitol treatment [16, 23], which has been shown to rescue α-DG functional glycosylation in FKRP^P448L^ mice. Nevertheless, this promising result needs further investigation to determine if this beneficial effect can be extended to other *FKRP* mutations, and if ribitol treatment presents any detrimental side effects. In any case, most efforts to date have focused on gene therapy by delivering fully functional FKRP via adeno-associated virus (AAV) to muscle cells of *FKRP*-mutant mouse models [17–20]. These studies have shown improvement of the dystrophic phenotype upon injections of AAV expressing FKRP under the control of systemic or muscle specific promoters. However, this approach has raised two major concerns, the efficacy of FKRP delivery, which was shown to decrease with age [20], and potential dose-dependent toxicity, suggesting that *FKRP* expression levels may need to be controlled [19]. In a combined gene/cell therapy study, Frattini and colleagues overexpressed FKRP in satellite cells isolated from *FKRP*-mutant mice. Transplantation of these engineered satellite cells into FKRP dystrophic mice led to the rescue of α-DG functional glycosylation, which the authors hypothesized to be due to the diffusion of FKRP via exosomes from the injected cells [21]. Even though the mechanism of rescue focused on the exosome delivery of FKRP, it remains to be determined the nature of engraftment, meaning clear identification and characterization of engrafted donor-derived myofibers.

In the context of cell therapy, pluripotent stem cells are particularly attractive due to their unlimited proliferative capacity and ability to differentiate into all cell types, allowing for the generation of large numbers of myogenic progenitors endowed with in vivo regenerative potential, as shown by transplantation studies using several mouse models of MD [24–27]. To determine whether LGMD2I associated with *FKRP* mutations could benefit from the transplantation of pluripotent stem cell-derived myogenic progenitors, here we generated an immunodeficient *FKRP*-mutant mouse model and validated this through the transplantation of mouse and human pluripotent stem cell-derived myogenic progenitors. Our data show robust engraftment that is accompanied by restoration of α-DG functional glycosylation and amelioration of disease phenotypes, thus supporting the therapeutic benefit of cell transplantation for LGMD2I and potentially other FKRP-associated muscle disorders.

## Methods

### Cell culture

Inducible (i) Pax3-GFP mouse embryonic stem (ES) cells were differentiated as previously described [24, 28], with the exception that ES cells were maintained in 1:1 ES medium and 2 inhibitors (2i) medium and in the absence of a mouse embryonic feeder layer. ES medium consists of KnockOut^TM^ DMEM (Invitrogen) supplemented with 15% FBS (Embryomax FBS, Millipore), 1% penicillin-streptomycin (Invitrogen), 2 mM Glutamax (Gibco), 0.1 mM non-essential amino acids (Gibco), and 0.1 mM β-mercaptoethanol (Gibco). 2i medium consists of neurobasal medium (Invitrogen) and DMEM F12 medium (Invitrogen) supplemented with 0.5% N2 (Life Technologies), 0.5% B27 (Life Technologies), 0.05% BSA (Sigma), 1% penicillin-streptomycin (Invitrogen), 150 μM monothioglycerol (MP Biomedicals), 3 μM GSK3β inhibitor (CHIR 990217; Tocris), 1 μM PD 0325901 (Cayman), and 1000 U/ml LIF (Millipore). For future in vivo tracking, iPax3 ES cells were labeled with lentiviral vector encoding the fusion protein histone 2B-red fluorescent protein (H2B-RFP; LV-RFP plasmid, Addgene #26001). Briefly, LV-RFP was co-transfected with packaging plasmids Δ8.91 and pVSVG into 293 T cells using the LTX transfection reagent (Thermo Fisher Scientific). Lentiviral-containing supernatant was collected 48 h later, filtered, and used to transduce iPax3 ES cells using the spin infection method (90 min at 2500 rpm at 30 °C). RFP+ ES cells were purified by FACS and maintained in ES + 2i medium.

For the transplantation of human cells, we used PAX7-induced myogenic progenitors since these have been extensively characterized in our laboratory for their in vivo regenerative potential [27, 29–31]. For this, a human wild-type induced pluripotent stem (iPS) cell line was transduced with doxycycline-inducible iPAX7-IRES-GFP lentivirus, as previously described [29]. Transduced iPS cells were cultured in suspension for 2 days to derive embryoid bodies, which were further cultured in medium supplemented with 10 μM GSK3β inhibitor (CHIR99021; Tocris) for 2 days, followed by treatment with 200 nM BMP inhibitor (LDN193189; Cayman Chemical) and 10 μM TGFβ inhibitor (SB431542, Cayman Chemical) for 2 days to derive somitic mesoderm-like cells. At day 5, cells were treated with 1 μg/ml doxycycline (dox) to induce PAX7 expression. Embryoid bodies were then plated onto gelatin-coated dishes in the presence of dox and bFGF (5 ng/ml) to derive a monolayer of cells. Four days later, these were dissociated and purified by FACS based on GFP expression to purify for PAX7+ myogenic progenitors, which were maintained in culture for up to 3-4 passages in the presence of dox and bFGF (Fig. S2d) [32].

### Generation and characterization of FKRP^P448L^-NSG mice

All animal studies were performed according to protocols approved by the University of Minnesota Institutional Animal Care and Use Committee. The FKRP^P448L^ mouse model [33] was obtained from Jackson Laboratories, where this strain was backcrossed to generate a congenic C57BL/6 (B6). To generate an immunodeficient FKRP^P448L^ mouse model, we have crossed the FKRP^P448L^ mutant onto the NSG (NOD/SCID; IL2 receptor gamma) background. These mice lack all functional classes of lymphocytes. F1 males (carrying gamma-c, which is X-linked) were backcrossed, and N1 pups carrying *FKRP* mutations and homozygous for NOD/SCID and IL2Rg were identified by PCR. To confirm immunodeficiency, peripheral blood was collected from the facial vein of B6, FKRP^P448L^, and FKRP^P448L^-NSG mice, stained with anti-mouse CD3e PE (145-2C11), anti-mouse CD19 PE-Cy7 (1D3), anti-mouse NK1.1 FITC (PK136), and anti-mouse pan-NK cells (DX5) antibodies, and analyzed by flow cytometry.

### Cell transplantation and muscle collection

Prior to transplantation, mice were anesthetized with ketamine/xylazine at 80 mg/kg by intraperitoneal (IP) injection. Cell transplantation was performed in tibialis anterior (TA) muscles of 3-5 weeks FKRP^P448L^-NSG or FKRP^P448L^ mice that had been pre-injured or not with cardiotoxin (CTX, 15 μl of 10 μM stock; Latoxan). Myogenic progenitors were injected at 1 × 10^6^ (resuspended in 15 μl of PBS) using a 22 g Hamilton syringe. As control, the contralateral leg was injected with 15 μL of PBS. For the transplantation of immunocompetent FKRP^P448L^ mice, recipients received intraperitoneal (IP) injections of the immunosuppressant agent tacrolimus (MedChemExpress) at a dose of 5 mg/kg. Treatment began 2 days before transplantation and ended by the day of euthanasia [29]. Engraftment of mouse cells was assessed at 4 weeks (short-term) and 5 months (long-term) post-transplantation. Engraftment of human cells was assessed at 6 weeks post-transplantation (short-term).

### Immunofluorescence staining

Muscles were embedded in Tissue-Tek O.C.T. compound (Sakura), and cryomolds containing embedded tissues were snap frozen on isopentane pre-cooled with liquid nitrogen. Cryosections of 14 μm were collected on glass slides, and prior to staining, rehydrated with PBS for 5 min at room temperature. Both cultured cells and muscle cryosections were fixed for 30 min at room temperature with 4% PFA, washed with PBS, permeabilized with 0.3% Triton X100 (Sigma) in PBS for 15 min at room temperature, washed again with PBS, blocked for 30 min blocking with 3% BSA (Sigma), and subsequently incubated with primary antibodies overnight at 4 °C. Primary antibodies included Pax7 (mouse 1:10, DSHB), IIH6C4 (IIH6, mouse IgM 1:200, 05-593 Millipore), laminin α2 (Lam, rat 1:200, Sc-59854 Santa Cruz), RFP (rabbit 1:500, ab62341 Abcam), dystrophin (Dys, mouse 1:20, DYS1-CE Leica), human LAMIN A/C (rabbit 1:500, ab108595 Abcam), and human DYSTROPHIN (DYS, mouse 1:50, MANDYS106, DSHB). The following day, samples were rinsed with PBS and then incubated with Alexa Fluor (Thermo Fisher Scientific) secondary antibodies and 4,6-Diamidino-2-phenylindole (DAPI, Santa Cruz) for 1 h at room temperature. After washing three times with PBS, sections were dried and mounted with Prolong Gold with DAPI (Invitrogen). Samples were analyzed by confocal microscopy (NikonNiE C2 upright confocal microscope). Image processing and quantification were performed with the Fiji software. H&E staining was performed as described [34].

Merge images of DAPI, IIH6, and RFP or LAMIN A/C were used to quantify donor-derived fibers. A total of 10-12 cryosections, separated by approximately 460 μm, were analyzed for the quantification of donor-derived myofibers. For the quantification of donor-derived satellite cells, merge images of laminin α-2, RFP, Pax7, and DAPI were used to quantify the proportion of Pax7+/RFP- and Pax7+/RFP+ cells. For the quantification of centrally nucleated myofibers, we used merge images of dystrophin, IIH6, RFP, and DAPI.

### Western blot

For biochemical analysis, TA muscles were snap frozen in liquid nitrogen, pulverized in a liquid nitrogen cooled mortar and pestle, and resuspended in lysis buffer Tris-buffer saline (TBS, 50 mM Tris-HCl, pH 7.5, 150 mM NaCl) with 1% Triton X-100 (Sigma) and a cocktail of protease inhibitors (Complete, Millipore). Lysates were placed on an end over rotator for 30 min at 4 °C and then centrifuged at 13000 rpm for 30 min at 4 °C. The supernatant containing the protein extract was collected, and protein concentration was determined using Bradford assay (Sigma). Briefly, 100 μg of protein lysates were loaded in each lane, resolved in SDS–PAGE, transferred to PVDF membranes (Immobilon-P; Millipore), and blocked 1 h in PBS, 0.1% Tween®20 (Sigma), and 5% milk (RPI). Membranes were incubated overnight at 4 °C with primary antibodies: IIH6 (1:1000, 05-593 Millipore) and β-DG (1:1000; MANDAG2 DSHB). After incubation with infrared fluorescent secondary antibodies (Li-cor and Thermo Fischer Scientific), membranes were visualized with Licor’s Odyssey® Infrared Imaging System. Image processing and quantification were performed with the Image studio software.

### Laminin overlay assay

The laminin binding assay was performed as previously described [35], with minor modifications. Briefly, 150 μg of protein was separated on 4–15% SDS polyacrylamide gels by electrophoresis and then transferred to PVDF membranes (Immobilon-P; Millipore). Membranes were blocked 1 h with PBS and 5% dry milk (RPI) at room temperature, rinsed with TBS (50 mM Tris-HCl, pH 7.5, 150 mM NaCl), and incubated for 2 h at room temperature in TBS containing 3% BSA, 1 mM CaCl_2_, 1 mM MgCl_2_ and 1 mg/ml native laminin (1:1000, L2020 Sigma). Membranes were washed twice for 10 min in TBSS (TBS containing 1 mM CaCl_2_ and 1 mM MgCl_2_) and incubated overnight at 4 °C with TBSS and anti-laminin (1:1000, L9393 Sigma). Afterwards, membranes were washed twice 10 min with TBSS. Then, incubated with the secondary antibody: anti-rabbit 680 (1:10000; 355569 Invitrogen) for 1 h at room temperature, washed twice for 10 min with TBSS and visualized using Licor’s Odyssey® Infrared Imaging System.

### Muscle preparation for mechanical studies

For the measurements of contractile forces, mice were anesthetized with avertin (225-250 mg/kg by IP injection) and intact TA muscles were dissected and placed in an experimental organ bath filled with mammalian Ringer solution containing 120.5 mM NaCl, 20.4 mM NaHCO_3_, 10 mM dextrose, 4.8 mM KCl, 1.6 mM CaCl_2_, 1.2 mM MgSO_4_, 1.2 mM NaH_2_PO_4_, and 1.0 mM pyruvate. Each chamber was perfused continuously with 95% O_2_– 5% CO_2_ and maintained at a temperature of 25 °C. Muscles were stimulated by an electric field generated between two platinum electrodes placed longitudinally on either side of the muscle (Square wave pulses 25 V, 0.2 ms in duration, 150 Hz). Three minutes of recovery period were allowed between stimulations. Specific force (sFo) was determined by normalizing maximum isometric tetanic force to cross section area (CSA) which were obtained by dividing the muscle mass (mg) by the product of muscle length (mm) and 1.06 mg/mm^3^ the density of skeletal muscle [24].

### Statistical analysis

Differences between two groups were assessed by using the Student’s *t* test or the Mann-Whitney *U* test (force measurement). Differences between multiple groups were assessed by ANOVA. Statistical analyses were performed using the Prism Software (GraphPad). *p* values < 0.05 were considered significant.

## Results

### Generation of an immunodeficient *FKRP*-mutant mouse model for LGMD2I

**Fig. 1:**
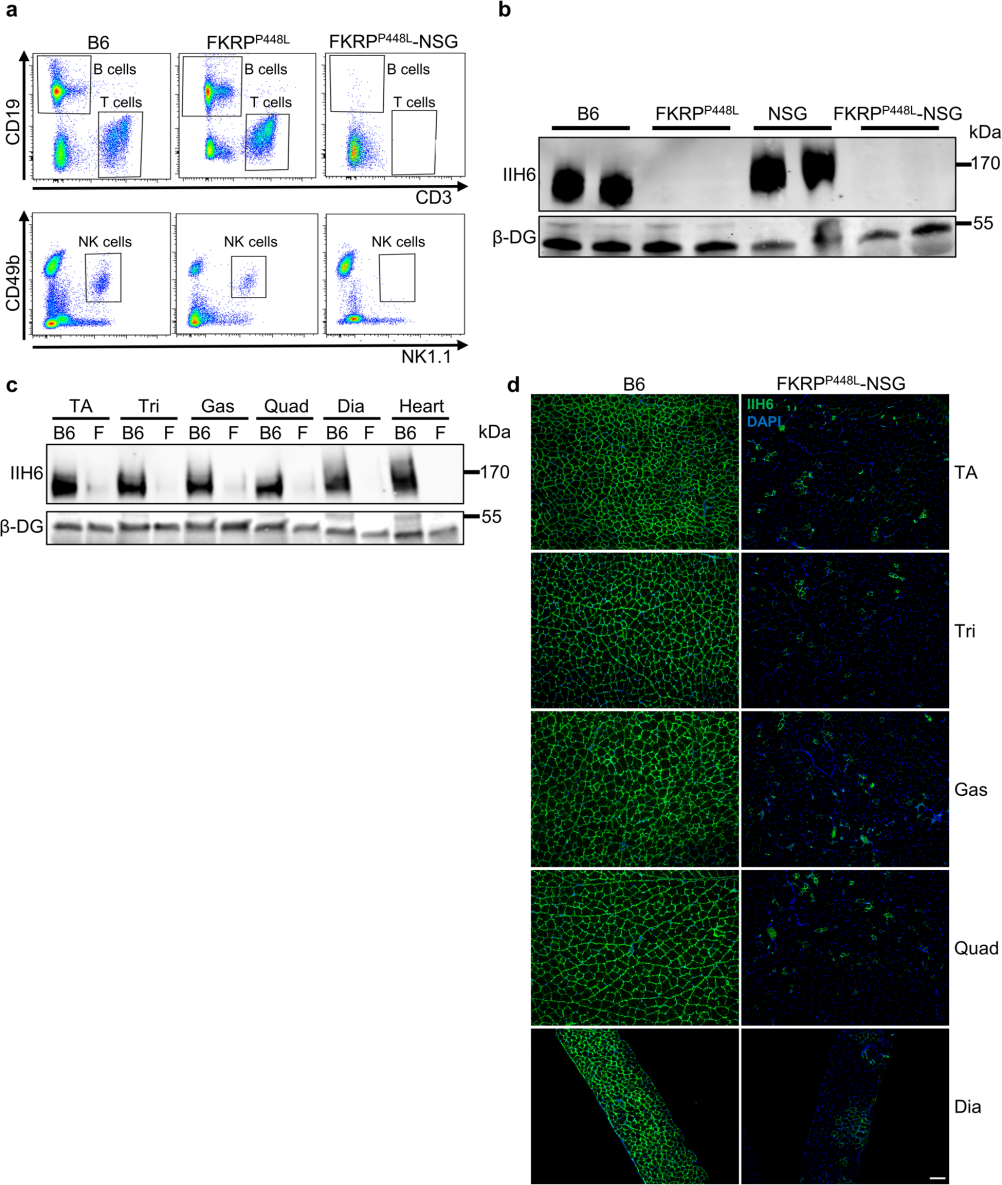
Characterization of FKRP^P448L^-NSG mice. **a** Representative plots show FACS profile for B (CD19), T (CD3), and NK (CD49b/NK1.1) cells in the peripheral blood of B6 (control), FKRP^P448L^, and FKRP^P448L^-NSG mice. **b** Western blot for IIH6 in TA muscle lysates from 10-week-old B6, FKRP^P448L^, NSG, and FKRP^P448L^-NSG mice. β-DG was used as loading control. **c** Western blot for IIH6 in TA, triceps (Tri), gastrocnemius (Gas), quadriceps (Quad), diaphragm (Dia), and heart lysates from 7-week-old B6 and FKRP^P448L^-NSG mice. β-DG was used as loading control. **d** Representative images show IIH6 immunostaining in TA, Tri, Gas, Quad, and Dia muscles from 7-week-old B6 and FKRP^P448L^-NSG mice. DAPI stained nuclei. Scale bar is 100 μm

To provide a receptive environment for the transplantation of mouse and human cells, we generated an immunodeficient *FKRP*-mutant mouse model by crossing FKRP^P448L^ mice [33] with the NSG strain (NOD/SCID; IL2 receptor common gamma chain) [36, 37]. Once we obtained FKRP^P448L^-NSG mice homozygous at all loci, the peripheral blood of these mice was analyzed by FACS, which confirmed the depletion of B, T, and NK cells in the FKRP^P448L^-NSG mouse model, whereas samples from control B6 and FKRP^P448L^ mice contained all these three lymphocyte subsets (Fig. 1a). Western blot analysis using the IIH6 antibody, which is specific to the α-DG laminin binding domain [3, 4], confirmed the lack of functionally glycosylated α-DG in FKRP^P448L^-NSG mice, similarly to their immunocompetent counterparts (Fig. 1b).

Previous literature [38] and our own findings (data not shown) indicate that background staining with the IIH6 antibody may become an issue as the *FKRP*-mutant mice get older (> 2-3 months), thus potentially interfering with proper engraftment assessment. An alternative to circumvent this hurdle would be to transplant younger FKRP^P448L^-NSG mice at 3 weeks of age, which would allow for engraftment assessment under 2 months of age. Of note, histological assessment of TA muscles from 7-week-old FKRP^P448L^-NSG mice confirmed dystrophic phenotype, as shown by the presence of centrally nucleated myofibers (Fig. S1a, b). To verify the feasibility of this transplantation timing, we assessed IIH6 immunoreactivity in several muscles of 7-week-old FKRP^P448L^-NSG mice by western blot and immunofluorescence staining. As shown in Fig. 1 c and d, relatively low levels of α-DG functional glycosylation were detected, and therefore 3-week-old FKRP^P448L^-NSG mice were used for the transplantation studies described here.

### Transplantation into pre-injured muscles of FKRP^P448L^-NSG mice

**Fig. 2:**
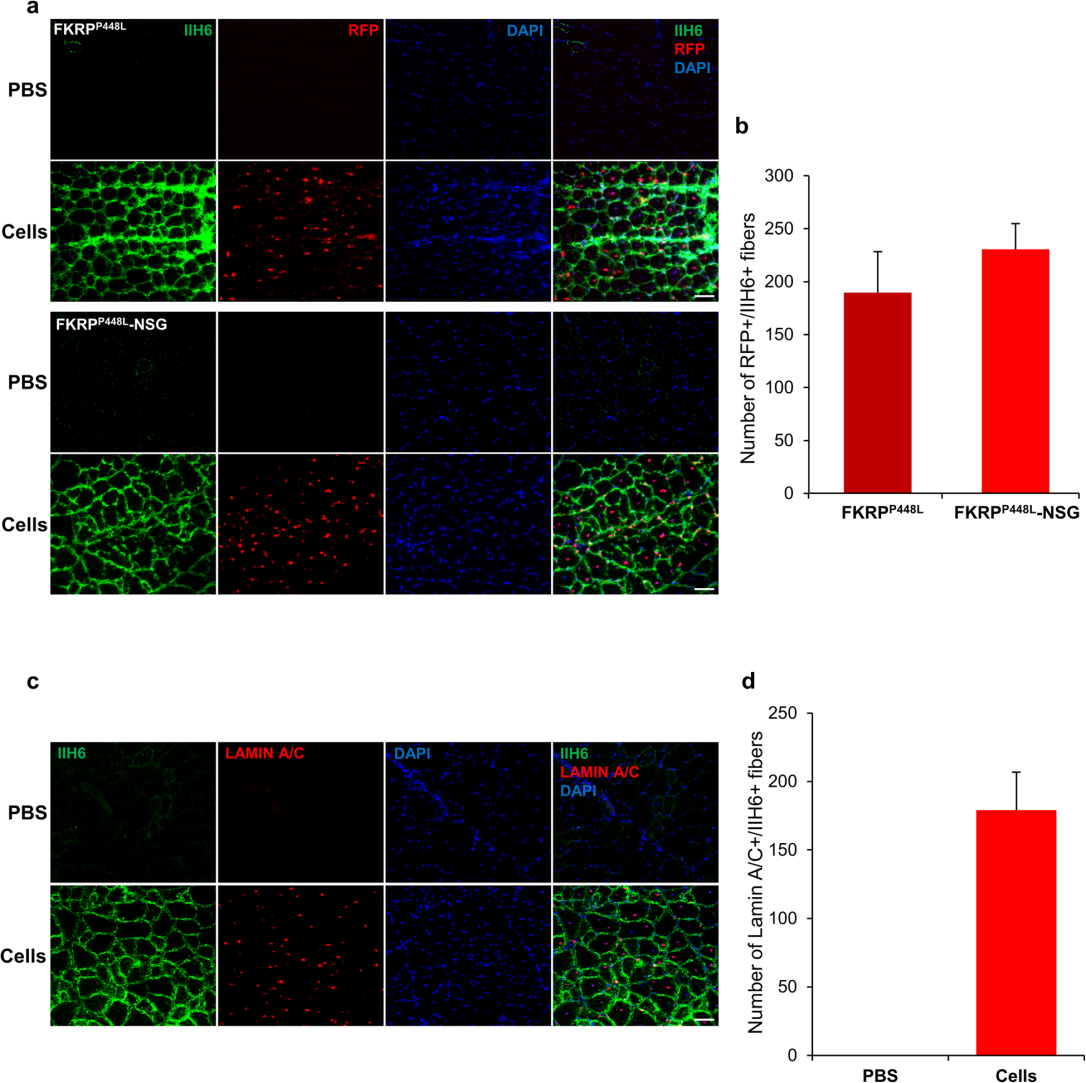
Validation of FKRP^P448L^-NSG mice as a model for transplantation studies. **a** Representative images show immunostaining for IIH6 (in green) and RFP (in red) in muscle sections from FKRP^P448L^ (upper panel) and FKRP^P448L^-NSG (lower panel) mice that had been pre-injured with CTX and injected with mouse ES cell-derived myogenic progenitors in the right leg and PBS in the contralateral leg. DAPI stained nuclei (in blue). Scale bar is 50 μm. **b** Engraftment quantification based on the number of RFP+/IIH6+ myofibers (from **a**). Data are shown as mean + SEM (*n* = 4; 4 females for FKRP^P448L^, and 2 males and 2 females for FKRP^P448L^-NSG). **c** Representative images show immunostaining for IIH6 (in green) and human LAMIN A/C (in red) in muscle sections from FKRP^P448L^-NSG that had been injected with human iPS cell-derived myogenic progenitors (lower panel) or PBS (upper panel). DAPI stained nuclei (in blue). Scale bar is 50 μm. **d** Engraftment quantification based on the number of IIH6+/LAMIN A/C+ myofibers (from **c**). Data are shown as mean + SEM from 2 independent cohorts (*n* = 8; 6 males and 2 females)

To facilitate in vivo tracking, we began transplantation studies using mouse ES cell-derived myogenic progenitors expressing the fusion protein H2B-RFP **(**Fig. S2a, b). Importantly, H2B-RFP genetic manipulation did not interfere with the capacity of these cells to express functionally glycosylated α-DG, as shown by IIH6 immunoreactivity in H2B-RFP-labeled ES cell-derived myotubes (Fig. S2c). H2B-RFP-labeled ES cell-derived myogenic progenitors were injected directly into the TA muscles of FKRP^P448L^-NSG mice. In this cohort, TA muscles were pre-injured with CTX 24 h prior to cell transplantation. As control, we also transplanted immunocompetent FKRP^P448L^ mice, which were treated daily with the immunosuppressive agent tacrolimus. Engraftment was assessed 4 weeks later by immunostaining with IIH6 and RFP antibodies, which clearly revealed the presence of donor-derived RFP+ myofibers that were also positive for IIH6 (Fig. 2a). Similar levels of engraftment were observed in both FKRP^P448L^-NSG and tacrolimus-treated FKRP^P448L^ mice (Fig. 2b).

Having validated the novel immunodeficient FKRP^P448L^-NSG model, we next assessed the ability of human iPS cell-derived myogenic progenitors to engraft in the FKRP^P448L^-NSG model. Six weeks after transplantation, human engraftment was determined by immunostaining using IIH6 in combination with an antibody specific to human LAMIN A/C. As expected, we did not detect myofibers positive for human LAMIN A/C and IIH6 in PBS-injected muscles (Fig. 2c, upper panels). On the other hand, evident human donor-derived myofiber contribution was observed in muscles that had been transplanted with human iPS cell-derived myogenic progenitors, as shown by the presence of myofibers positive for both LAMIN A/C and IIH6 (Fig. 2c, lower panels). Quantification showed approximately 200 double-positive myofibers (Fig. 2d). Human specific DYSTROPHIN and PAX7 immunostaining confirmed the human origin of the LAMIN A/C+ fibers and their regenerative capacity (Fig. S3). These results confirm the usefulness of this model for human cell transplantation.

### Pre-injury is not required to enable efficient engraftment in FKRP^P448L^-NSG mice

**Fig. 3:**
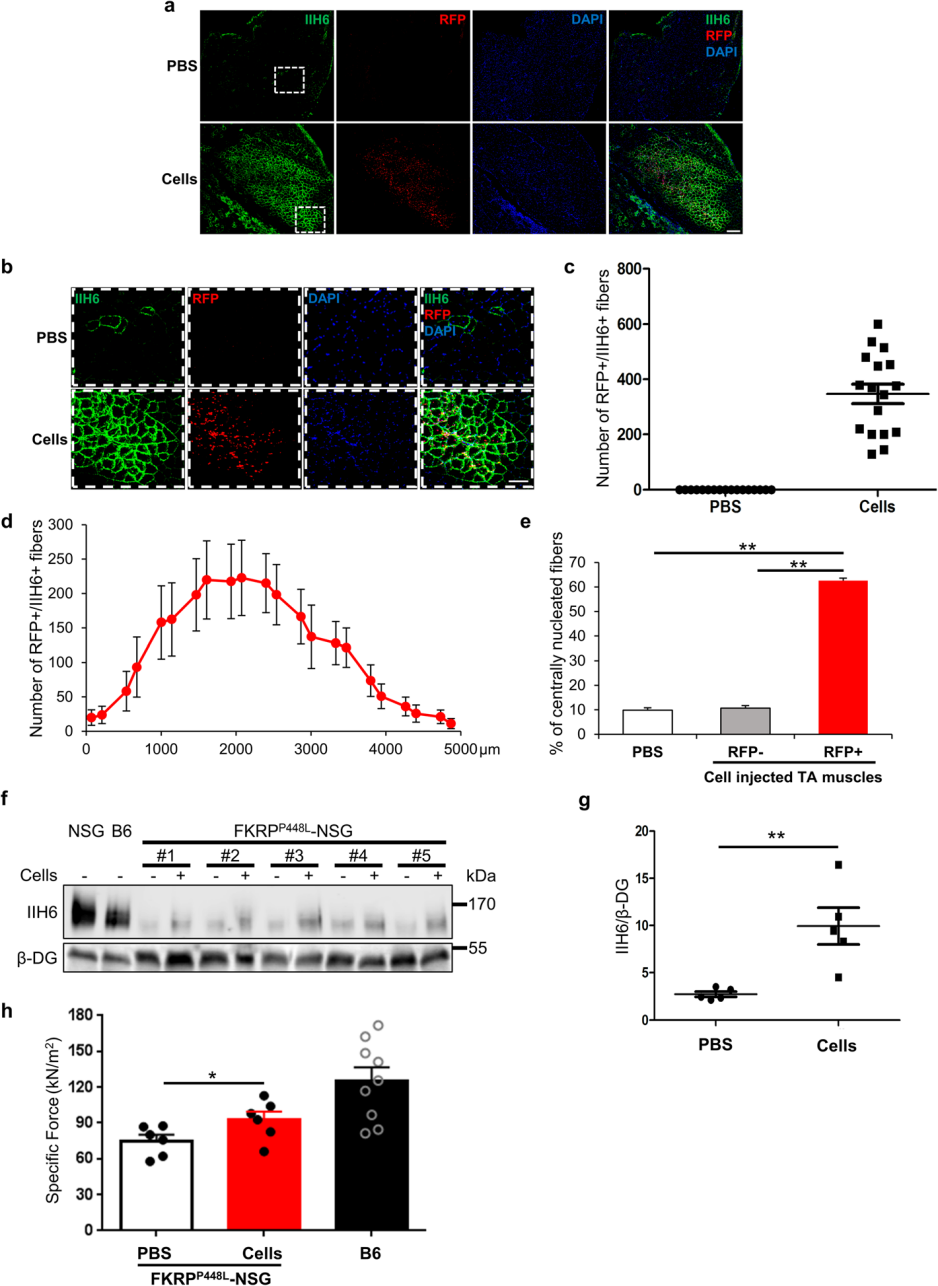
Engraftment analysis upon cell transplantation in non-injured muscles. **a** Representative images, capturing the whole engraftment area, show immunostaining for IIH6 (in green) and RFP (in red) in TA muscles from non-injured FKRP^P448L^-NSG that had been injected with PBS (upper panel) or mouse ES cell-derived myogenic progenitors (lower panel). DAPI stained nuclei (in blue). Scale bar is 200 μm. **b** High magnification image of donor-derived engrafted myofibers (from **a**, white square). Scale bar is 100 μm. **c** Engraftment quantification based on the number of RFP+/IIH6+ myofibers. Data are shown as mean + SEM from three different cohorts (*n* = 17; 9 males and 8 females). **d** Distribution of the number of RFP+/IIH6+ myofibers along the TA muscle (*n* = 7). **e** Quantification of the percentage of centrally nucleated myofibers in the PBS injected TA muscles, RFP- and RFP+ area of the cell injected TA muscles. Data are shown as mean + SEM (*n* = 4; 2 males and 2 females). **p < 0.01. **f** Representative western blot for IIH6 in TA lysates from 7-week-old FKRP^P448L^-NSG mice that have been injected at 3-weeks of age with mouse ES cell-derived myogenic progenitors or PBS (contralateral muscle as negative control) (*n* = 5; 2 males and 3 females). B6 and NSG muscles were used as reference. β-DG was used as a loading control. **g** Respective quantification of IIH6 band intensity. **e** Normalized with β-DG. Data are shown as mean + SEM. **p < 0.01. **h** Effect of cell transplantation on specific (*sF*_*0*_: F_0_ normalized to CSA) force. Data are shown as mean + SEM (*n* = 6; 3 males and 3 females). B6 mouse TA muscles were used as a reference (*n* = 8, 2 males and 6 females). *p < 0.01

To determine whether pre-injury is required for muscle engraftment in the FKRP^P448L^-NSG mouse model, next we transplanted mouse ES cell-derived myogenic progenitors into non-injured TA muscles. An average of 350 donor-derived RFP+/IIH6+ myofibers were quantified per TA muscle among 17 FKRP^P448L^-NSG recipient mice from 3 different cohorts (Fig. 3a-c). To examine the distribution of donor-derived myofibers, in another cohort of 7 recipient mice, we quantified engraftment along the length of transplanted TA muscles (approximately 4500 μm). Consistent engraftment was detected along the length of the muscle (Fig. 3d), showing the ability of myogenic progenitors to distribute well within transplanted muscles. Quantification of centrally nucleated myofibers revealed that approximately 60% of donor-derived myofibers were centrally nucleated at 4 weeks post-transplantation (Fig. 3e). Similar engraftment was observed following the transplantation of these cells into non-injured muscles of tacrolimus-treated immunocompetent FKRP^P448L^ mice (Fig. S4). In addition, to better assess the overall rescue of α-DG functional glycosylation in the whole TA muscle, we normalized the area positive for both RFP and IIH6 to the total section area (Fig. S5a). This analysis revealed about 15% restoration in the levels of α-DG functional glycosylation in transplanted TA muscles (Fig. S5b). This rescue was consistent along the muscle, as shown by the quantification of the area containing functionally glycosylated α-DG (Fig. S5b). To verify whether human myogenic progenitors are also able to promote regeneration in the absence of pre-injury, we transplanted human iPAX7 iPS cell-derived myogenic progenitors in non-injured TA muscles of 3-week-old FKRP^P448L^-NSG mice. As shown in Fig. S6a-d, human donor-derived myofibers are also observed in the absence of pre-injury but at lower levels when compared to mouse transplanted counterparts (127.83 ± 19.94 vs*.* 346.76 ± 35.39, respectively; Fig. S6b, c), probably due to the xeno nature of this transplantation.

To further validate the rescue of α-DG functional glycosylation, we performed western blot analysis in mice that had been transplanted with mouse ES cell-derived myogenic progenitors. We determined the linear range of detection for IIH6 and β-DG antibodies with different amounts of total protein and observed that 100 ug of total protein from FKRP^P448L^-NSG cells injected TA lysates would be adequate for quantification (Fig. S7a-c). As shown in Fig. 3 f and g, high levels of α-DG functional glycosylation were detected in TA muscles that had been transplanted with myogenic progenitors. This finding was further confirmed with two additional independent transplantation cohorts (Fig. S7d), and corroborated by the laminin-overlay assay. Consistently with IIH6 data, superior laminin binding was observed in transplanted muscles (Fig. S7e, f). Importantly, using isolated muscle force measurements, we observed improvement in muscle strength in transplanted TA muscles when compared to PBS-injected controls (Fig. 3h).

### Donor-derived satellite cell engraftment and long-term regeneration

**Fig. 4:**
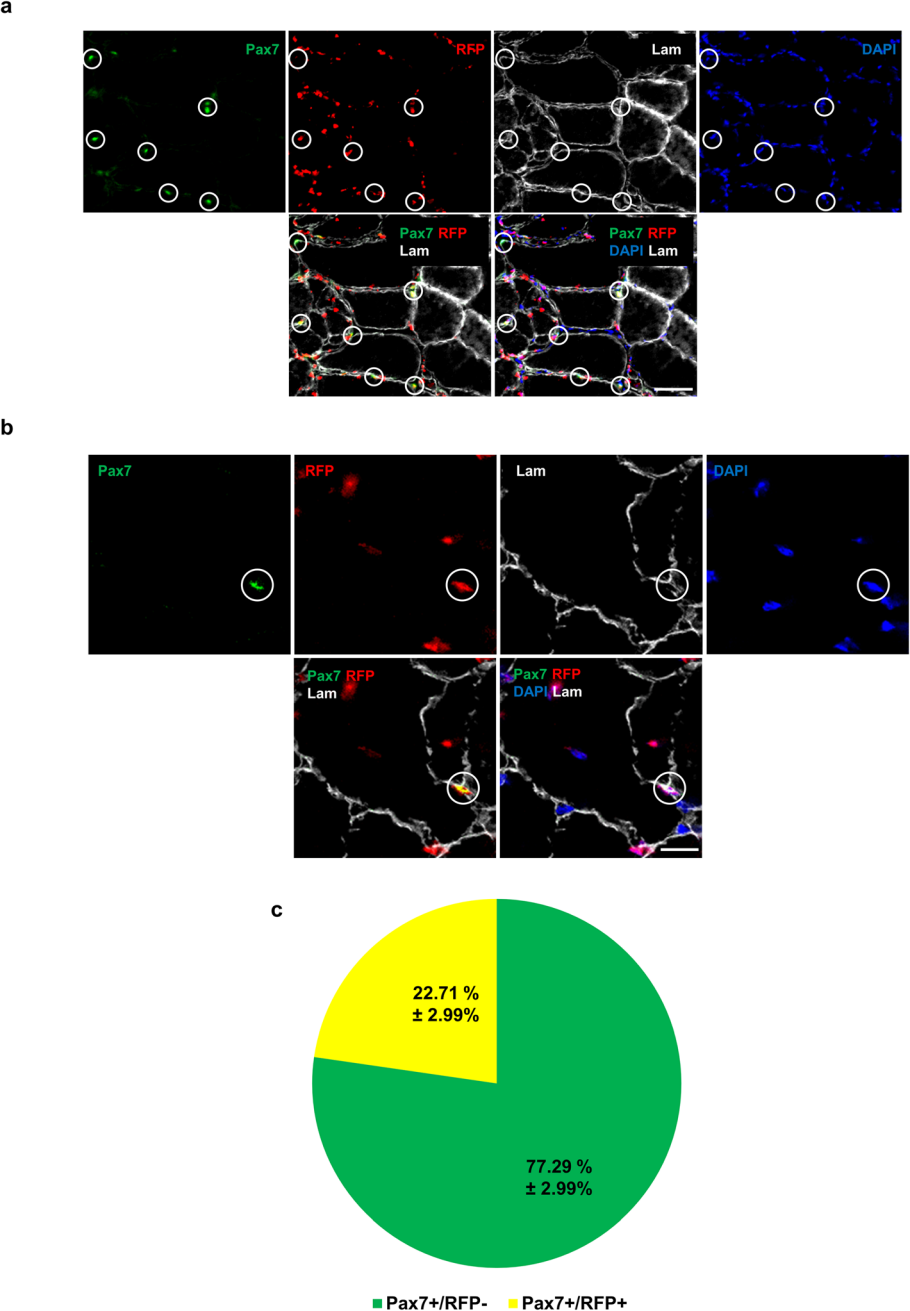
Donor-derived satellite cell engraftment. **a**, **b** Representative images show satellite cell staining from non-injured FKRP^P448L^-NSG TA muscles transplanted with mouse ES cell-derived myogenic progenitors. Circles show cells double positive for Pax7 (green) and RFP (red) under the basal lamina (lam in gray), indicating donor-derived satellite cells. Nuclei in blue. Scale bar is 50 μm (a) and 20 μm (b). **c** Percentage of Pax7+/RFP+ cells and Pax7+/RFP- cells per muscle sections. Data are shown as mean + SEM (*n* = 8; 5 males and 3 females)

Next, we investigated whether pluripotent stem cell-derived myogenic progenitors have the ability to seed the satellite cell compartment following their transplantation into FKRP^P448L^-NSG mice. This is critical to ensure the long-term repopulation potential of transplanted cells. To address this question, we stained muscle sections with antibodies to Pax7 (to identify satellite cells), RFP (to distinguish donor contribution), and laminin-α2 (to confirm sub-laminal localization). Our results show that transplanted myogenic progenitors are able to populate the satellite pool, as shown by the presence of Pax7+/RFP+ nuclei identified under the basal lamina (Fig. 4a, b). Quantification of engrafted areas revealed that approximately 20% of Pax7+ cells were also positive for RFP, denoting significant donor contribution to the satellite cell compartment (Fig. 4c).

**Fig. 5:**
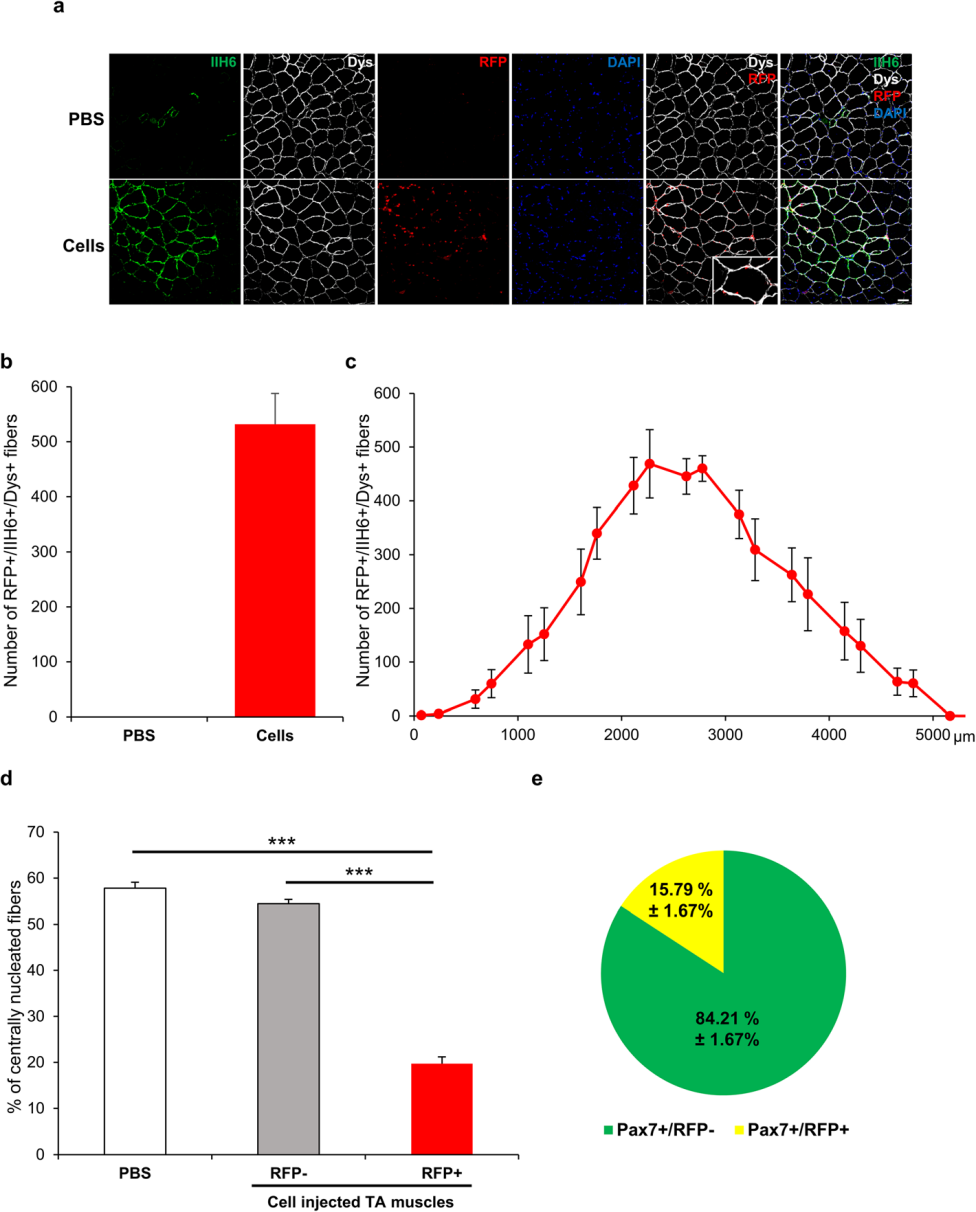
Assessment of long-term engraftment in FKRP^P448L^-NSG recipients. **a** Representative images show immunostaining for IIH6 (in green), Dys (in white), and RFP (in red) in non-injured TA muscles from FKRP^P448L^-NSG mouse collected 4 months after transplantation. For each recipient, one TA muscle was injected with PBS (upper panel) and the contralateral TA with mouse ES cell-derived myogenic progenitors (lower panel). DAPI stained nuclei (in blue). Scale bar is 50 μm. **b** Engraftment quantification based on the number of RFP+/IIH6+/Dys+ myofibers. Data are shown as mean + SEM (*n* = 7; 3 males and 4 females). **c** Distribution of the number of RFP+/IIH6+/Dys+ myofibers along the TA muscle (*n* = 7; 3 males and 4 females). **d** Quantification of the percentage of centrally nucleated myofibers in the PBS injected TA muscles, RFP- and RFP+ area of the cell injected TA muscles. Data are shown as mean + SEM (*n* = 7 from a). ***p < 0.001. **e** Percentage of Pax7+/RFP+ cells and Pax7+/RFP- cells per muscle sections. Data are shown as mean + SEM (*n* = 7; 3 males and 4 females)

The high percentage of donor-derived satellite cells suggests that transplanted cells may be endowed with long-term regenerative capacity. To test this, we assessed long-term engraftment in a cohort of FKRP^P448L^-NSG mice that had been injected with cells at 3 weeks of age in the absence of injury. Immunohistological analysis and subsequent quantification confirmed the presence of donor-derived myofibers at 4 months post-transplantation (Fig. 5a-c). Of note, we observed that persistence of engraftment was accompanied by reduction in the percentage of centrally nucleated myofibers (~ 20%; Fig. 5d) when compared to results from short-term transplantation (~ 60%; Fig. 3e). Quantification of RFP+ satellite cells in this long-term cohort showed the persistence of donor-derived RFP+/Pax7+ cells, thus suggesting maintenance of satellite cell engraftment (Fig. 5e). These results confirm the long-term regenerative potential of mouse ES cell-derived myogenic progenitors in the LGMD2I mouse model, and the amelioration of dystrophic pathology.

## Discussion

Here we generated an immunodeficient *FKRP*-mutant mouse model, which allowed us to test the effectiveness of mouse and human cell transplantation to rescue α-DG functional glycosylation in the context of LGMD2I. We show that pluripotent stem cell-derived myogenic progenitors engraft robustly in this mouse model, and that engraftment results in restoration of functionally glycosylated α-DG. Extensive characterization of engraftment levels revealed a maximum of 600 donor-derived myofibers, contributing to up to 30% rescue of α-DG functional glycosylation. Biochemical assessment, including western blot to IIH6 and laminin binding assay, corroborated functional rescue of α-DG functional glycosylation. Importantly, muscle strength was improved in engrafted muscles, and long-term studies demonstrated persistence of myofiber and satellite cell engraftment.

An interesting and unexpected finding of the present study was the robust and widespread engraftment observed in the FKRP^P448L^ mouse model in the absence of muscle pre-injury (in both immunodeficient and immunocompetent background). Due to the relatively low turnover in skeletal muscle, strategies to induce muscle injury prior to transplantation, such as CTX, barium chloride, cryoinjury, or irradiation, are commonly used to enhance the muscle regenerative response and therefore, better assessment of the repopulation potential of a given cell population [24, 39, 40]. This applies not only to non-disease mice but also to mouse models of MDs, such as the *mdx* for Duchenne MD [24, 41–43] and the *α-sarcoglycan null* for LGMD2D [44], among others. Attesting this, Vallese and colleagues have shown superior muscle engraftment following the transplantation of human myoblasts in cryoinjured recipient muscles when compared to non-injured counterparts [45]. In the context of pluripotent stem cell-derived myogenic progenitors, we have documented that their transplantation into non-injured muscles of *mdx* mice results in more limited engraftment, which was also more restricted to the injection site, opposed to CTX-injured muscles [24]. Of note, previous studies have shown that pre-injury is not required for the engraftment of these myogenic progenitors in dystrophin/utrophin double knockout mice [26, 46], probably due to the much more severe dystrophic phenotype characteristic of this Duchenne MD mouse model [47].

Even though further studies would be required to understand the mechanisms underlining the enhanced regenerative response of transplanted cells in the absence of pre-injury in FKRP^P448L^-NSG and immunocompetent FKRP^P448L^ dystrophic mice. These findings are highly relevant for pre-clinical studies since they better represent the scenario of a clinical trial aimed at cell-based therapy for MD patients, which evidently would not make use of such pre-injury procedures.

## Conclusions

Using a newly generated immunodeficient FKRP-mutant mouse model, we have shown that transplanted pluripotent stem cell-derived myogenic progenitors are able to engraft, rescue α-DG functional glycosylation, and improve muscle strength, providing proof-of-concept for the potential therapeutic application of stem cell therapy for LGMD2I associated with FKRP mutations.

## Supplementary information

**Additional file 1: Supplementary Figure S1.** Histological characterization of FKRP^P448L^-NSG mice. a) Representative images show H&E staining in TA muscle cryosections from 7-week-old B6 (control) and FKRP^P448L^-NSG mice. Arrows indicate centrally located nuclei and asterisks denote the presence of infiltrating mononuclear cells. Scale bar is 50 μm. **b)** Quantification of the percentage of centrally nucleated myofibers in the TA muscles of 7-weel-old B6 and FKRP^P448L^-NSG mice. Data are shown as mean + SEM (TA muscles from 4 mice). ***p < 0.001. **Supplementary Figure S2.** Mouse ES cell labelling/differentiation and human iPS cell differentiation. a) Outline representing the labeling of iPax3-GFP mouse ES cells with the H2B-RFP encoding lentivirus and subsequent myogenic differentiation. **b)** Representative FACS plots show percentage of RFP+ cells at different stages of differentiation: left: ES cells, center: embryoid bodies (EBs) before sorting, and right: myogenic progenitors used for transplantation (P4). **c)** Representative images show immunostaining for IIH6 and RFP in myotubes resulting from the *in vitro* differentiation of ES cells. IIH6, RFP, and nuclei are shown in green, red and blue, respectively. Scale bar 50 μm. **d)** Outline representing the timeline of myogenic differentiation of human iPAX7 iPS cells. **Supplementary Figure S3.** Characterization of human engraftment. a) Representative images show immunostaining for human DYSTROPHIN (in gray) and human LAMIN A/C (in red) in muscle sections from CTX-injured FKRP^P448L^-NSG mouse TA muscles that had been injected with human iPS cell-derived myogenic progenitors or PBS (from Fig. 2c). DAPI stained nuclei (in blue). Scale bar is 100 μm. **b)** Representative images show satellite cell staining in the TA muscles described in (**a**). Circles show cells double-positive for PAX7 (green) and LAMIN A/C (red) under the basal lamina (Lam in gray) indicating donor-derived satellite cells. Nuclei in blue. Scale bar is 50 μm. **c)** High magnification image of donor-derived satellite cell. Scale bar is 20 μm. **Supplementary Figure S4.** Engraftment analysis in non-injured muscles of FKRP^P448L^ immunocompetent mice. **a**) Representative images show immunostaining for IIH6 (in green) and RFP (in red) in non-injured TA muscles from FKRP^P448L^ mice that had been injected with PBS (upper panel) or mouse ES cell-derived myogenic progenitors (lower panel). DAPI stained nuclei (in blue). Scale bar is 100 μm. **b)** Engraftment quantification based on the number of RFP+/IIH6+ myofibers (from **a**). Data are shown as mean + SEM (n = 5; 2 males and 3 females). **c)** Distribution of the number of RFP+/IIH6+ myofibers along the TA muscle (n = 5; 2 males and 3 females). **Supplementary Figure S5.** Engrafted area quantification in non-injured muscles of FKRP^P448L^-NSG mice. **a**) Representative image used to assess the size of the engrafted area (marked in red) compared to the total cryosection area (marked in blue). IIH6 (gray) and RFP (red) allow the delimitation of the area of engraftment. Scale bar is 500 μm. **b)** Distribution along the length of TA muscle of the percent engraftment (RFP+/IIH6+) area. Data are shown as mean + SEM (n = 7; 4 males and 3 females). **Supplementary Figure S6.** Engraftment analysis in non-injured muscles transplanted with human iPS cells. **a**) Representative images show immunostaining for IIH6 (in green) and human LAMIN A/C (in red) in muscle sections from non-injured FKRP^P448L^-NSG mouse TA muscles that had been injected with human iPS cell-derived myogenic progenitors (lower panel) or PBS (upper panel). DAPI stained nuclei (in blue). Scale bar is 50 μm. **b)** Engraftment quantification based on the number of IIH6+/LAMIN A/C+ myofibers (from **a**). Data are shown as mean + SEM (n = 6, 4 males and 2 females). **c)** Distribution of the number of IIH6+/LAMIN A/C+ myofibers along the TA muscle (n = 6; 4 males and 2 females). **d)** Representative images show immunostaining for human DYSTROPHIN (in gray) and human LAMIN A/C (in red) in muscle sections from non-injured FKRP^P448L^-NSG mouse TA muscles injected with iPS cell-derived myogenic progenitors or PBS (from **a)**. DAPI stained nuclei (in blue). Scale bar is 50 μm. **Supplementary Figure S7.** Additional western blot analysis and Laminin overlay assay. **a**) Western blot for IIH6 and β-DG in TA lysates from 7-week-old FKRP^P448L^-NSG mice (2 TA muscles pooled) that had been injected at 3-weeks of age with mouse ES cell-derived myogenic progenitors. To determine the linear range of detection for IIH6 and β-DG antibodies, an increasing amount of protein (0, 25, 50, 100, 125, 150, 200 μg) was loaded. **b)** Quantification of IIH6 band intensity according to the amount of protein loaded. **c)** Quantification of the β-DG band intensity related to the amount of protein loaded. **d)** Western blot for IIH6 in TA lysates from 7-week-old FKRP^P448L^-NSG mice that had been injected at 3-weeks of age with mouse ES cell-derived myogenic progenitors or PBS (contralateral muscle as negative control). Data from two independent experiments (n = 5 for each), and their respective quantification of IIH6 band intensity normalized with β-DG. Data are shown as mean + SEM. *p < 0.01. **e)** Detection of laminin binding. No calcium served as negative control. **f)** Quantification of laminin band intensity normalized with β-DG. Data are shown as mean + SEM (n = 4; 2 males and 2 females). *p < 0.01.

## Data Availability

The datasets used and/or analyzed during the current study are available from the corresponding author on reasonable request. Materials used in this study are commercially available.

## Abbreviations

DG: Dystroglycan
FKRP: Fukutin-related protein
LGMD2I: Limb girdle muscular dystrophy 2I
MD: Muscular dystrophy
ECM: Extracellular matrix
AAV: Adeno-associated virus
i: Inducible
ES: Embryonic stem
2i: 2 inhibitors
H2B-RFP: Histone 2B-red fluorescent protein
iPS: Induced pluripotent stem
dox: Doxycycline
B6: C57BL/6
TA: Tibialis anterior
IP: Intraperitoneal injection
CTX: Cardiotoxin
IIH6: IIH6C4
DAPI: 4,6-diamidino-2-phenylindole
TBS: Tris-buffer saline
Tri: Triceps
Gas: Gastrocnemius
Quad: Quadriceps
Dia: Diaphragm
Lam: Laminin-α2
Dys: Dystrophin
DYS: Human DYSTROPHIN
EB: Embryoid body
