# Supplementary material for: Efficient engraftment of pluripotent stem cell-derived myogenic progenitors in a novel immunodeficient mouse model of limb girdle muscular dystrophy 2I
Source: Skelet Muscle. 2020 Apr 22;10:10. doi: 10.1186/s13395-020-00228-3 (PMC7175515; doi:10.1186/s13395-020-00228-3)
Supplement: Supplementary file 1 — Additional file 1: Supplementary Figure S1. Histological characterization of FKRPP448L-NSG mice. a) Representative images show H&E staining in TA muscle cryosections from 7-week-old B6 (control) and FKRPP448L-NSG mice. Arrows indicate centrally located nuclei and asterisks denote the presence of infiltrating mononuclear cells. Scale bar is 50 μm. b) Quantification of the percentage of centrally nucleated myofibers in the TA muscles of 7-weel-old B6 and FKRPP448L-NSG mice. Data are shown as mean + SEM (TA muscles from 4 mice). ***p < 0.001. Supplementary Figure S2. Mouse ES cell labelling/differentiation and human iPS cell differentiation. a) Outline representing the labeling of iPax3-GFP mouse ES cells with the H2B-RFP encoding lentivirus and subsequent myogenic differentiation. b) Representative FACS plots show percentage of RFP+ cells at different stages of differentiation: left: ES cells, center: embryoid bodies (EBs) before sorting, and right: myogenic progenitors used for transplantation (P4). c) Representative images show immunostaining for IIH6 and RFP in myotubes resulting from the in vitro differentiation of ES cells. IIH6, RFP, and nuclei are shown in green, red and blue, respectively. Scale bar 50 μm. d) Outline representing the timeline of myogenic differentiation of human iPAX7 iPS cells. Supplementary Figure S3. Characterization of human engraftment. a) Representative images show immunostaining for human DYSTROPHIN (in gray) and human LAMIN A/C (in red) in muscle sections from CTX-injured FKRPP448L-NSG mouse TA muscles that had been injected with human iPS cell-derived myogenic progenitors or PBS (from Fig. 2c). DAPI stained nuclei (in blue). Scale bar is 100 μm. b) Representative images show satellite cell staining in the TA muscles described in (a). Circles show cells double-positive for PAX7 (green) and LAMIN A/C (red) under the basal lamina (Lam in gray) indicating donor-derived satellite cells. Nuclei in blue. Scale bar is 50 μm. c) High magnifica [file 13395_2020_228_MOESM1_ESM.pptx]

## Slide 1
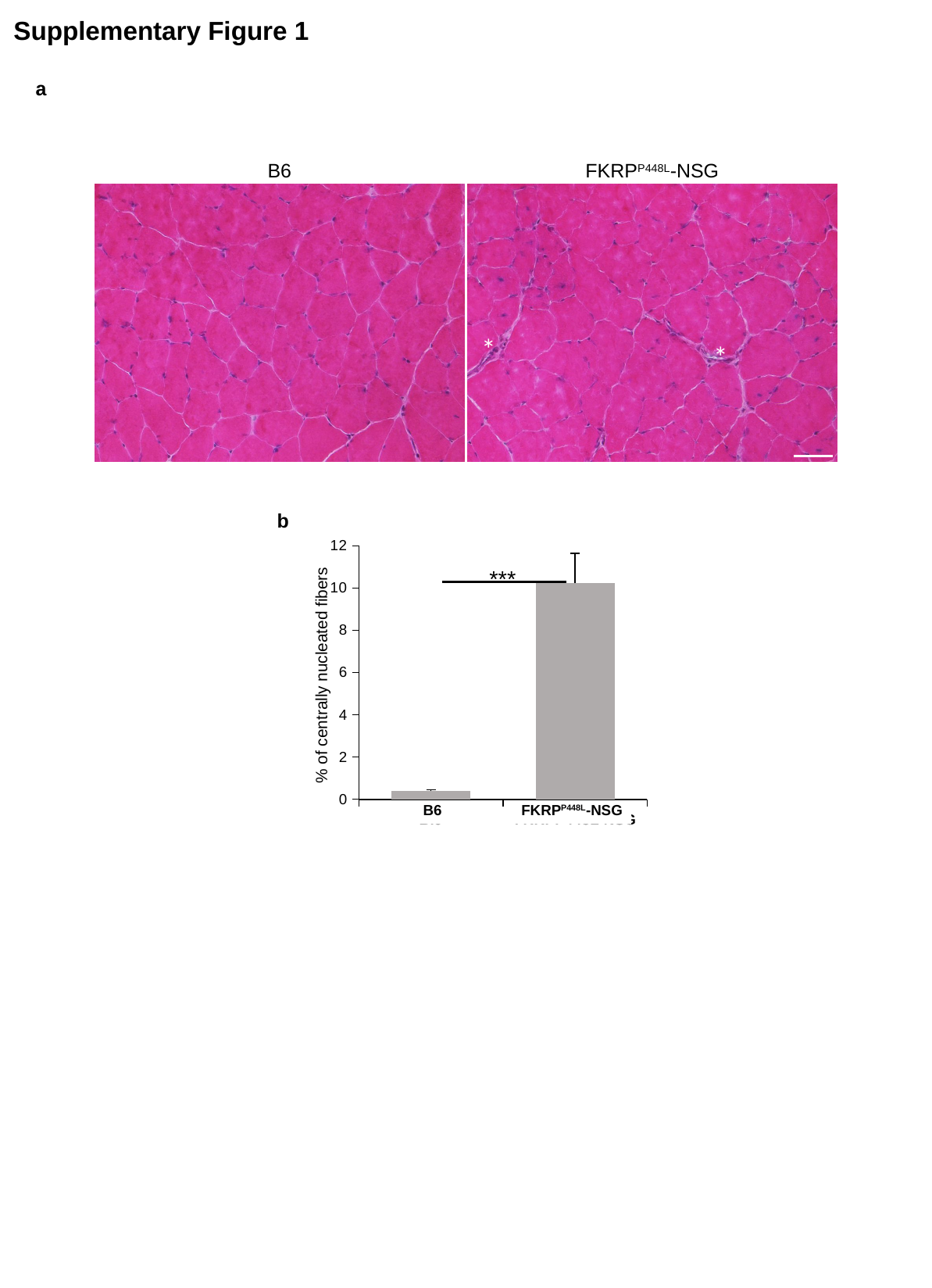

Supplementary Figure 1
a
B6
FKRPP448L-NSG
*
*
b
### Chart
| Category | |
|---|---|
| Bl6 | 0.3991787653156462 |
| FRRPP448L-NSG | 10.22326756992857 |% of centrally nucleated fibers
B6
FKRPP448L-NSG
***

## Slide 2
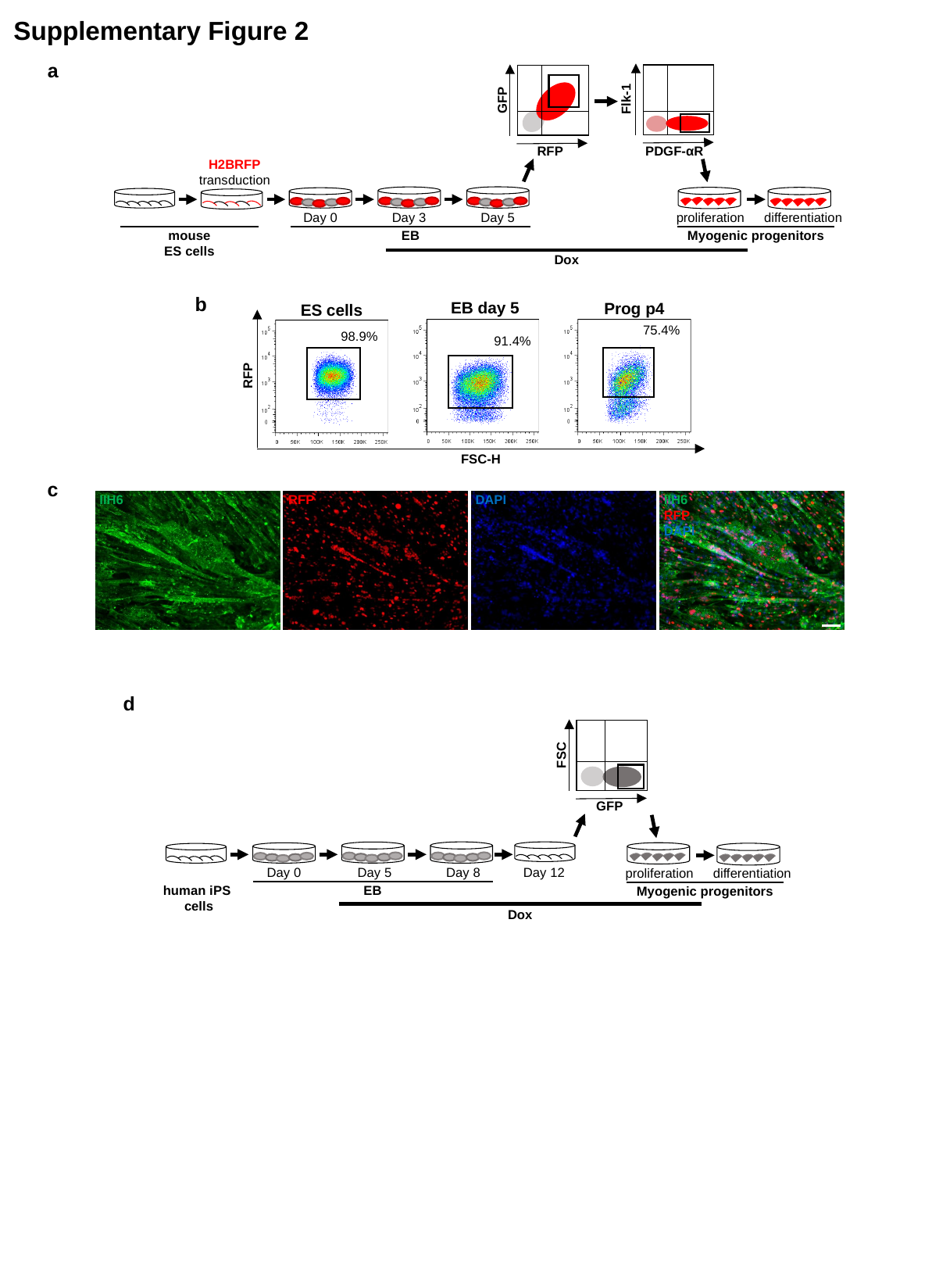

Supplementary Figure 2
a
Flk-1
GFP
RFP
PDGF-αR
H2BRFP transduction
Day 0
Day 3
Day 5
proliferation
differentiation
mouse
ES cells
EB
Myogenic progenitors
Dox
b
EB day 5
Prog p4
ES cells
91.4%
75.4%
98.9%
RFP
FSC-H
c
IIH6
RFP
DAPI
IIH6
RFP
DAPI
d
FSC
GFP
Day 0
Day 5
Day 8
Day 12
proliferation
differentiation
human iPS
cells
EB
Myogenic progenitors
Dox

## Slide 3
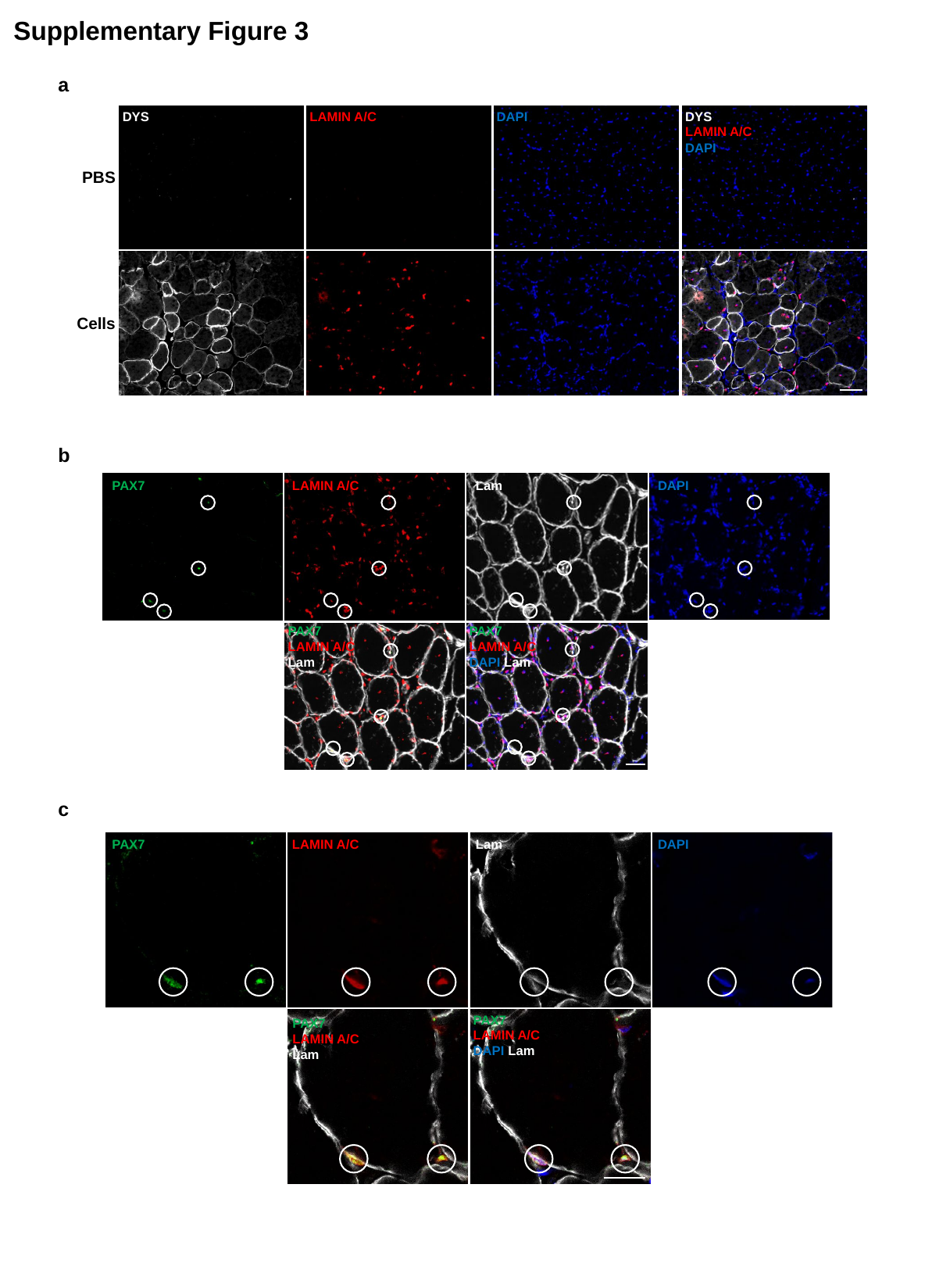

Supplementary Figure 3
a
DYS
LAMIN A/C
DAPI
DYS
LAMIN A/C
DAPI
PBS
Cells
b
PAX7
LAMIN A/C
Lam
DAPI
PAX7 LAMIN A/C Lam
PAX7 LAMIN A/C
DAPI Lam
c
PAX7
LAMIN A/C
Lam
DAPI
PAX7 LAMIN A/C
DAPI Lam
PAX7 LAMIN A/C Lam

## Slide 4
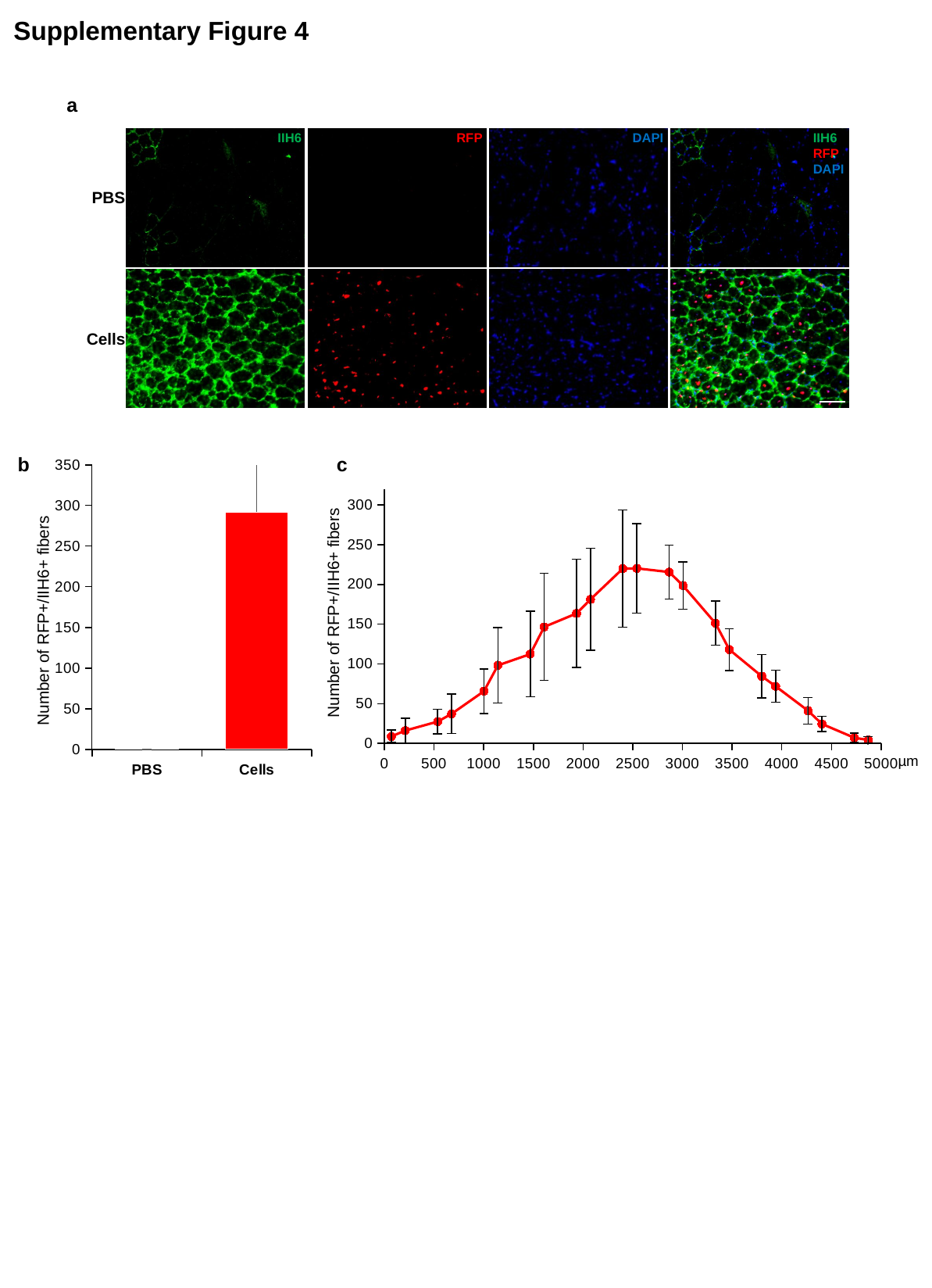

Supplementary Figure 4
a
IIH6
RFP
DAPI
IIH6
RFP
DAPI
PBS
Cells
b
c
### Chart
| Category | |
|---|---|
| PBS | 0.0 |
| Cells | 292.1666666666667 |
### Chart
| Category | |
|---|---|Number of RFP+/IIH6+ fibers
Number of RFP+/IIH6+ fibers
µm

## Slide 5
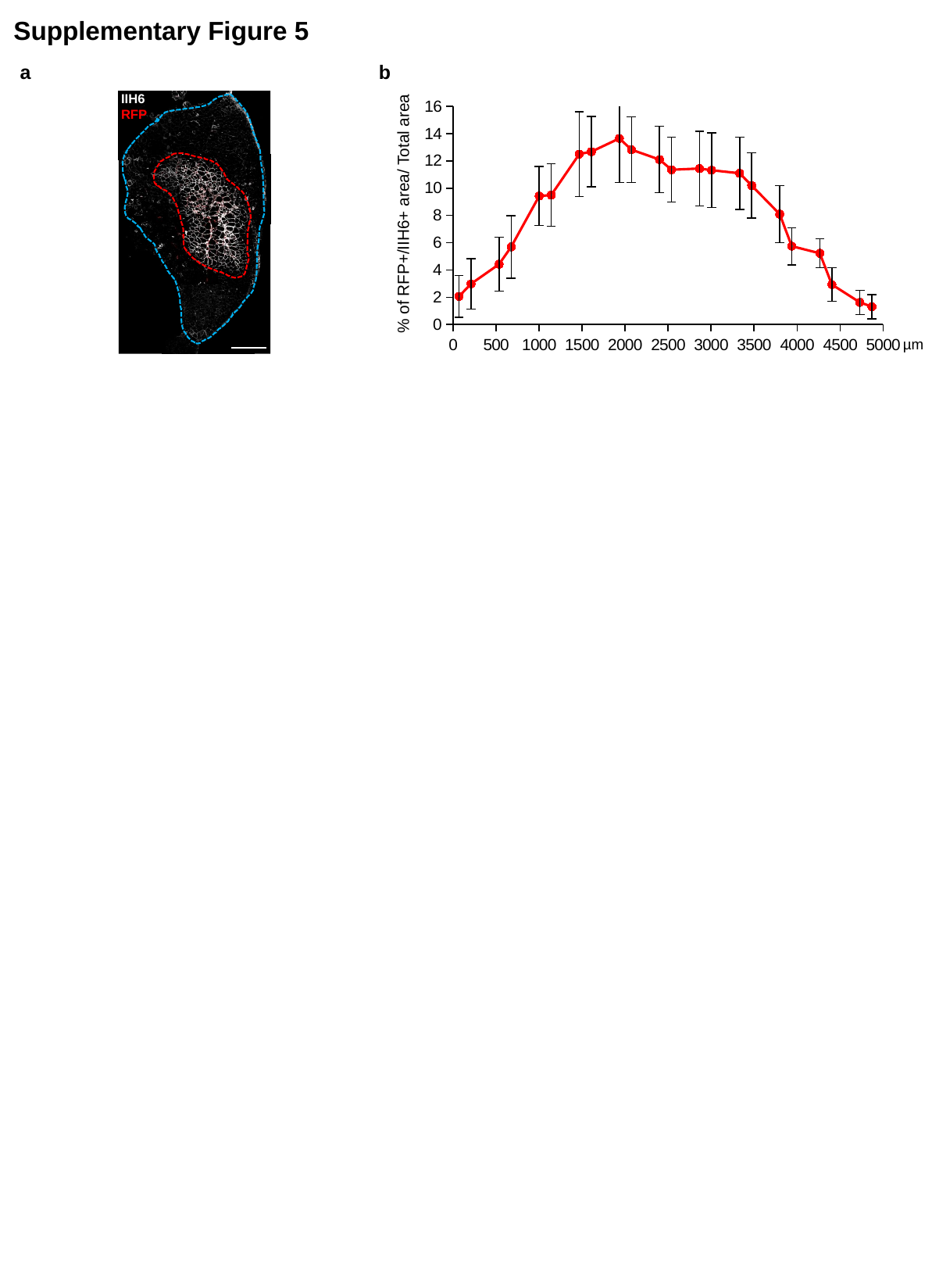

Supplementary Figure 5
a
b
IIH6
RFP
### Chart
| Category | average |
|---|---|% of RFP+/IIH6+ area/ Total area
µm

## Slide 6
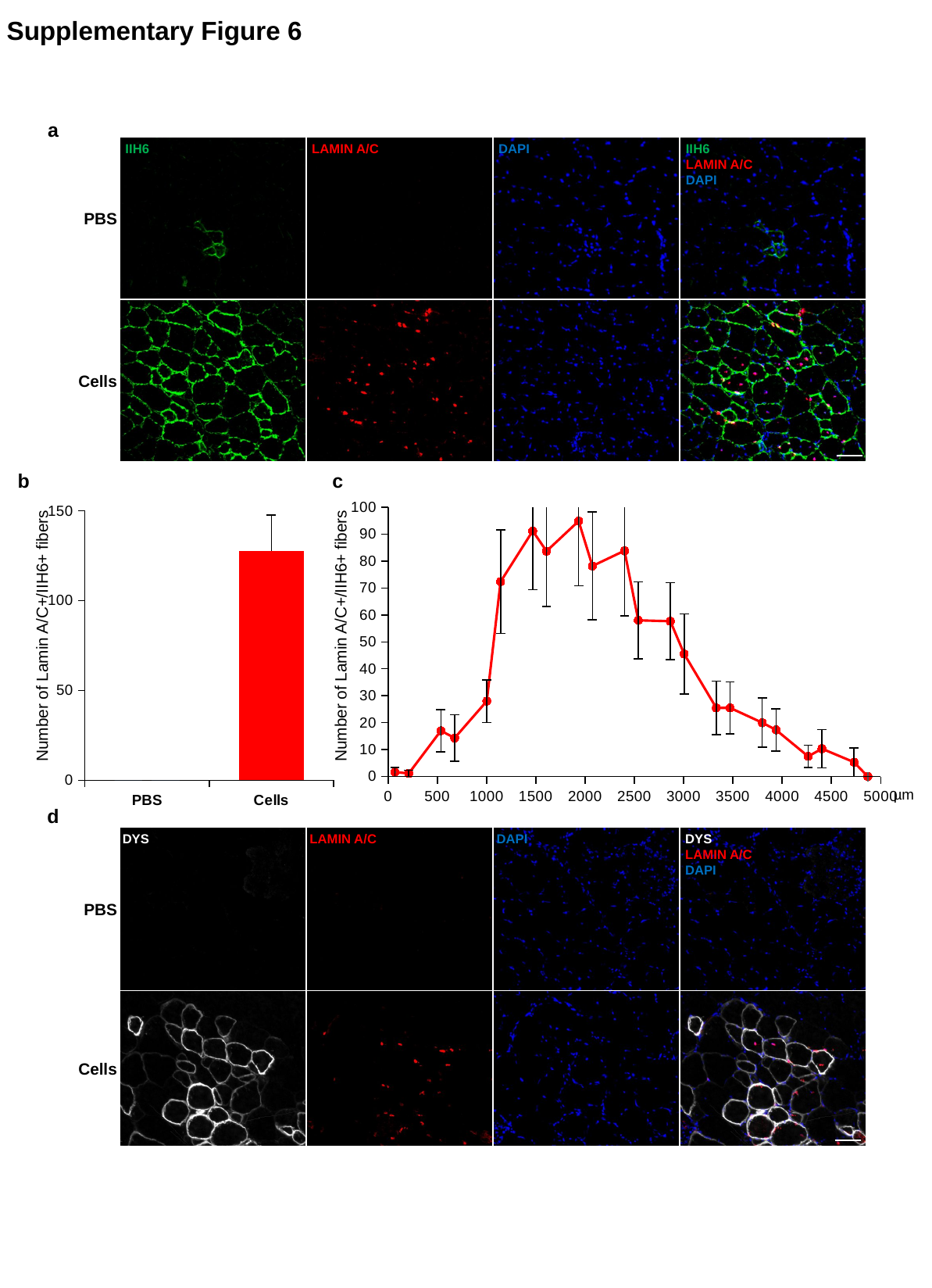

Supplementary Figure 6
a
IIH6
LAMIN A/C
DAPI
IIH6
LAMIN A/C
DAPI
PBS
Cells
b
c
### Chart
| Category | |
|---|---|
### Chart
| Category | |
|---|---|
| PBS | 0.0 |
| Cells | 127.83333333333333 |Number of Lamin A/C+/IIH6+ fibers
Number of Lamin A/C+/IIH6+ fibers
µm
d
LAMIN A/C
DYS
DAPI
DYS
LAMIN A/C
DAPI
PBS
Cells

## Slide 7
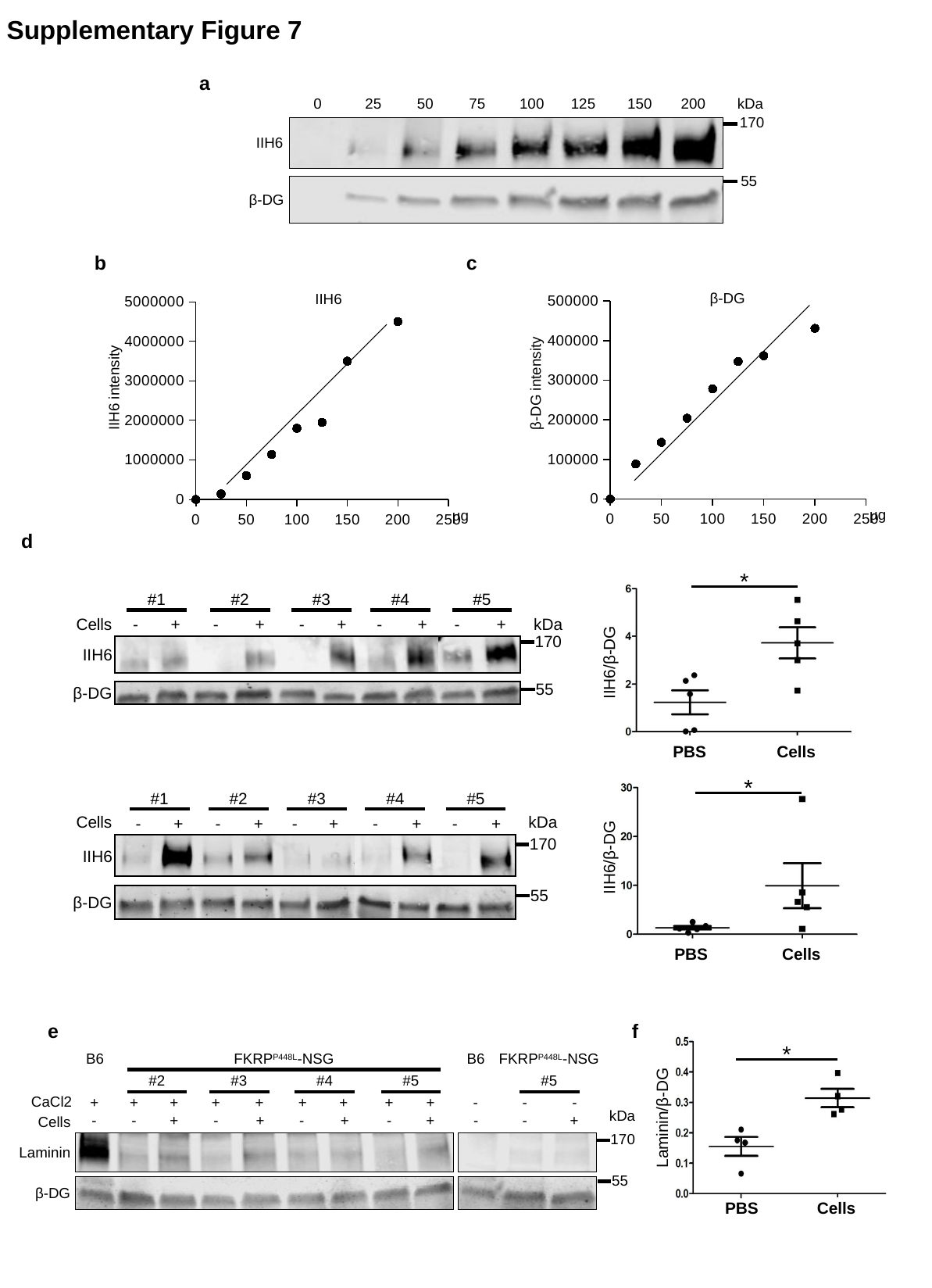

Supplementary Figure 7
a
0
25
50
75
100
125
150
200
kDa
170
IIH6
55
β-DG
b
c
β-DG
IIH6
### Chart
| Category | B dys |
|---|---|
### Chart
| Category | IIH6 |
|---|---|β-DG intensity
IIH6 intensity
µg
µg
d
*
#1
#2
#3
#4
#5
-
+
-
+
-
+
-
+
-
+
Cells
kDa
170
IIH6
55
β-DG
IIH6/β-DG
PBS
Cells
*
#1
#2
#3
#4
#5
Cells
kDa
-
+
-
+
-
+
-
+
-
+
170
IIH6
55
β-DG
IIH6/β-DG
PBS
Cells
e
f
*
B6
FKRPP448L-NSG
B6
FKRPP448L-NSG
#2
#3
#4
#5
#5
CaCl2
+
+
+
+
+
+
+
+
+
-
-
-
kDa
Laminin/β-DG
-
-
+
-
+
-
+
-
+
-
-
+
Cells
170
Laminin
55
β-DG
PBS
Cells
